# Quercetin Suppresses Human Glioblastoma Migration and Invasion via GSK3β/β-catenin/ZEB1 Signaling Pathway

**DOI:** 10.3389/fphar.2022.963614

**Published:** 2022-11-01

**Authors:** Bo Chen, Xiaoli Li, Lihong Wu, Duanfang Zhou, Yi Song, Limei Zhang, Qiuya Wu, Qichen He, Gang Wang, Xu Liu, Hui Hu, Weiying Zhou

**Affiliations:** ^1^ Department of Pharmacology, College of Pharmacy, Chongqing Medical University, Chongqing, China; ^2^ Chongqing Key Laboratory of Drug Metabolism, Chongqing Medical University, Chongqing, China; ^3^ Key Laboratory for Biochemistry and Molecular Pharmacology of Chongqing, Chongqing Medical University, Chongqing, China; ^4^ Chongqing Pharmacodynamic Evaluation Engineering Technology Research Center, Chongqing Medical University, Chongqing, China

**Keywords:** quercetin, metastasis, glioblastoma, epithelial-like mesenchymal transition, GSK3β/β-catenin/ZEB1 signaling

## Abstract

High invasiveness is a biological and clinical characteristic of glioblastoma and predicts poor prognosis of patients. Quercetin, a natural flavonoid compound, exhibits anticancer activity. However, we have a limited understanding of the possible underlying mechanism of quercetin in glioblastoma. In this study, we investigated the anticancer effect of quercetin in human glioblastoma cells. Our results showed that quercetin markedly suppressed the viability of glioblastoma cells *in vitro* and *in vivo*, and significantly inhibited glioblastoma cell migration and invasion. Moreover, quercetin reversed EMT-like mesenchymal phenotype and reduced the expression levels of EMT-related markers. Furthermore, we found that quercetin suppressed GSK-3β/β-catenin/ZEB1 signaling in glioblastoma. Taken together, our results demonstrate that quercetin inhibited migration and invasion of human glioma cells by suppressing GSK3β/β-catenin/ZEB1 signaling. Our study provides evidence that quercetin is a promising therapeutic natural compound to treat glioblastoma.

## 1 Introduction

Glioblastoma (GBM) is the most prevalent primary malignant intracranial brain tumors in adult (Alexander and Cloughesy, 2017; Molinaro et al., 2019). Owing to the highly aggressive and metastatic nature of GBM, although the “gold standard” therapeutic approaches (such as surgery, radiotherapy and chemotherapy and adjuvant temozolomide therapy) are usually used for GBM patients in clinical, the prognosis is still pathetic and median survival is only 15 months (Mckinnon et al., 2021). High invasiveness which makes it infiltrate into the surrounding soft tissues and difficult to be completely rooted out by surgery, is a main clinical distinguishing feature leading to relapse and death in GBM patients. Although tumor invasion has been widely concerned by many researchers, whereas the molecular mechanism of GBM invasion is still not completely clear. Consequently, a deeper understanding of the molecular mechanism of GBM invasion and developing effective treatment drugs with low toxicity are essential to GBM patients.

Epithelial-mesenchymal transformation (EMT), an important biological reversible process, refers to the transformation of epithelial-to-mesenchymal cells, which gives cells the ability to metastasize and invade. EMT not only plays a key role in the regulation of embryonic development, but also participates in tissue healing, organ fibrosis and cancer metastasis (Chen et al., 2017; Mittal, 2018). Most importantly, recently researches have revealed that mesenchymal phenotypes of cells are closely related to the high invasion ability of GBM (Lah et al., 2020). In the past decades, increasing evidences have proved that abnormally activated WNT signaling pathway leads to mesenchymal transition and is closely associated with glioma malignancy (Gong and Huang, 2012). WNT and β-catenin are highly expressed in tumor tissues of the patients with GBM, and it is associated with significantly shorter survival time (Xu et al., 2020). In recent years, targeting WNT/β-catenin signaling pathway has played a very important role in glioma therapy (Sandberg et al., 2013; Latour et al., 2021). In GBM, WNT/β-catenin signaling is activated and promotes tumor invasion by inducing expression of downstream EMT transcription factors like Twist, ZEB1, and Snail (Gong and Huang, 2012). Glycogen synthase kinase three beta (GSK3β) is a key molecule of the WNT/β-catenin signaling pathway and is a negative regulator of EMT (Domoto et al., 2020). Therefore, targeting GSK3β for GBM has attracted great attention due to activation of WNT/β-catenin-mediated proto-cancer pathways.

Natural compounds are known to have very good therapeutic effects in GBM, which are considered as promising therapies. In recent years, some studies report that Flavonoids, as a group of natural structures, such as luteolin, kaempferol and quercetin are a potential treatment measures to prevent or treat cancer *via* diverse mechanisms, such as apoptosis, autophagy, endoplasmic reticulum stress (Yang et al., 2020; Lee et al., 2021; Kusaczuk et al., 2022). Quercetin, widely distributed in plant kingdom, is a flavonol compound derived from a variety of fruits, vegetables and some Chinese medicine. As compared with other examined compounds, quercetin has low toxicity and various biological activities, such as strong anti-oxidant, anti-inflammatory, anti-viral and anti-cancer, which makes it an attractive chemical in the fight against diseases including cancer (Rauf et al., 2018; Tang et al., 2020; Eisvand et al., 2022). Importantly, a recent research has revealed that quercetin is a promising phytochemical for the treatment of GBM, which inhibits the progress of GBM through multiple mechanisms (Tavana et al., 2020). However, the potential molecular mechanism of quercetin against GBM invasion and metastasis is not fully understood. In this study, we aimed to determine whether quercetin could suppress GBM migration and invasion, and to investigate the possible underlying mechanism. We found that quercetin inhibited mesenchymal transition through GSK-3β/β-catenin/ZEB1signaling pathway *in vitro* and *in vivo*. Our data suggest that quercetin could be developed as a safe and high-efficiency therapeutic drug for human GBM.

## 2 Materials and methods

### 2.1 Reagents

Quercetin (purity ≥98.5%) and Temozolomide (TMZ) (purity ≥98%) were purchased from Aladdin (Shanghai, China) and dissolved in dimethyl sulfoxide (DMSO). Cell Counting Kit-8 was purchased from Bimake (Houston, TX, United States). Antibodies against GSK-3β, β-catenin, E-cadherin, N-cadherin, Vimentin, ZEB1, Akt, p-Akt and anti-rabbit IgG (Alexa Fluor^®^ 488 Conjugate) were purchased from Cell Signaling Technology (Danvers, MA, United States). Antibodies against β-actin, p-GSK-3β (Ser 9) were purchased from Santa Cruz (Dallas, Texas, United States). Horseradish peroxidase-conjugated goat anti-rabbit IgG, Horseradish peroxidase-conjugated goat anti-mouse IgG secondary antibodies and Phalloidin were purchased from Zhongshan Jinqiao Biotechnology Co. Ltd. (Beijing, China). Antibody against Ki67 was obtained from Affinity Biosciences (Zhenjiang, China). The SDS-PAGE Gel Quick Preparation Kit, RIPA lysis buffer, Crystal violet and BCA Protein Assay Kit were purchased from Beyotime Biotechnology (Shanghai, China). SB216763 was purchased from MedChemExpress (New Jersey, United States).

### 2.2 Cell culture

The human GBM cell lines U87MG and T98 were purchased from the cell bank of the Chinese Academy of Sciences (Shanghai, China). Primary mouse astrocytes a gift from Dr. Wenjun Li (Department of Pharmacy, The Third Affiliated Hospital of Chongqing Medical University, Chongqing, China). CHG-5 cell line was previously described (Li et al., 2016). All cell lines were cultured in Dulbecco’s Modified Eagle Medium (Hyclone, Logan, UT, United States) supplemented with 10% fetal bovine serum (Gibco, Grand Island, NY, United States) and 1% antibiotics (100 U/mL penicillin and 100 μg/ml streptomycin) in a humidified atmosphere with 5% CO_2_ at 37°C.

### 2.3 Cell viability assay

Cells were seeded in 96-well plates (3 × 10^4^/ml) and treated with 0, 10, 20, 40, 80, 160, 320 μM of Quercetin for 24, 48 h, 72 h respectively. CCK-8 (Cell Counting Kit-8) assay was performed using a standard protocol and optical density (OD) was read at 450 nm using a Synergy H1 microplate87 spectrophotometer (BioTek, Winooski, VT, United States). The cell survival rate was calculated relative to the control group.

### 2.4 Characterization of spontaneous movement using high content analysis

U87MG and CHG-5 cells were plated at 2.5 × 10^3^ per well in collagen-coated CellCarrier-96 Ultra microplate (6055708, PerkinElmer). After 12 h adherent culture, the cells were changed into serum-free medium and treated with the different concentrations of quercetin (20, 40, 80 μM) or vehicle. Then microplates were placed onto the pre-warmed Operetta system. Using Operetta’s automatic digital phase contrast algorithm, the digital phase contrast image of ×20 magnification (×20Air objective) for 12 h was obtained under the condition of imaging interval of 60 min. Migrating cells were tracked and imaged using automated single cell tracking algorithm of the Harmony 4.9 software (PerkinElmer).

### 2.5 Wound healing assay

U87MG and CHG-5 cells were cultured in six-well plates within culture medium containing 10% FBS and grown to nearly confluent cell monolayer. Then a linear wound was made by scratching the center of well with a 200 μl pipette tip. The monolayer cells were washed with PBS for twice to remove the detached cells and the remaining cells were maintained in the serum-free medium with or without quercetin (20, 40, 80 μM) for 12 h and 24 h. The gap distance of the wound was photographed by a light microscope (Nikon, Japan) and the area of the wound was measured.

### 2.6 Transwell migration assay

The migration capacity of U87MG and CHG-5 cells was evaluated by matrigel-coated transwell chamber (Corning, NY, United States). In short, cells were trypsinized, washed, and suspended in a serum-free medium with or without quercetin (20, 40, 80 μM) and seeded at a density of 5 × 10^4^ cells/well onto the upper chambers, the lower chambers were filled with culture medium added 10% FBS and the same concentration of quercetin as the upper chamber. After 24 h incubation, the cells on top surface of the chamber were rubbed off, while the cells on the lower surface were fixed with 4% para-formaldehyde for 30 min and stained with 5% crystal violet for 15 min. Then three random fields of every chamber were scanned to record the cells at the lower membrane side at ×200 magnifications using a microscope (Nikon, Japan), and calculated the migration rate of the two cancer cell lines.

### 2.7 Transwell invasion assay

The transwell chamber was pretreated with matrigel (Corning, NY, United States) and dried at 37°C for 1 h. Other procedures were the same as the one for the transwell migration assay. The results of the transwell invasion assay were also calculated according to the number of transferred cells.

### 2.8 Computer modeling and docking models of quercetin and Glioblastoma 3β

Protein molecular docking was analyzed using Discovery Studio 2016 software. The binding free energy was evaluated using CHARMM-based energies and a generalized Born model. The 3D structure of quercetin was uploaded to pharmMapper (http://www.lilab-ecust.cn/pharmmapper/). For the docking assay, the PDB files of proteins obtained from the RCSB PDB website (http://www.rcsb.org/) and the Moll file of quercetin obtained from the ZINC website (http://zinc.docking.org/) were imported into Discovery Studio 2016 to dock them together.

### 2.9 Western blotting analysis

Total cells samples were lysed with RIPA lysis buffer containing 1 mM protease inhibitor and 1 mM phosphatase inhibitor (Beyotime, Shanghai, China). The protein concentration was measured with BCA Protein Assay Kit. Subsequently, the protein was separated using SDS-PAGE and transferred to PVDF membranes. Membranes were blocked with 5% skim milk in TBST (10 mM Tris-HCl, 0.1 M NaCl, 0.1% Tween20, pH 7.4), and then incubated with primary antibodies overnight at 4°C. Then membranes were washed four times with TBST and then incubated with HRP-conjugated secondary antibodies at room temperature for 2 h. Then protein bands were examined with chemiluminescence imaging system (Tanon, Shanghai, China) using ECL substrate.

### 2.10 Immunohistochemistry assay

Tumor specimens were fixed in 4% paraformldehyde, and then embedded in paraffin and sectioned. The sections were deparaffinized/rehydrated. The sections were then immersed in citrate unmasking solution and heated in a microwave for antigen unmasking. After blocking with 50 μl of goat serum for 30 min at room temperature, the sections were incubated overnight with Ki67 antibody at 4°C, followed by incubation with HRP-conjugated secondary antibodies at room temperature for 2 h. The signal from each section was visualized with 3′-diaminobenzidine reagent and counterstained with hematoxylin. Images were captured using a light microscope (OLYMPUS).

### 2.11 Immunofluorescence assays

Cells were plated on cover slips and cultured in a 24-well plate. After treatment with quercetin, cells were fixed with 4% paraformaldehyde for 30 min at room temperature. The cells were washed with PBS three times, and then permeabilized with 0.3% Triton-X 100 for 10 min. Subsequently, the cells were blocked with QuickBlock™ immunostaining block solution for 30 min. Then, the cells were incubated with primary antibodies (β-catenin 1:200) overnight at 4°C. After washing with PBS, the cells were incubated with anti-rabbit IgG (Alexa Fluor^®^ 488 Conjugate) for 2 h. The nucleus was stained with DAPI and examined with Leica TCS SP8 confocal microscope at ×630 magnification. The images were processed by Leica Application Suite X software.

### 2.12 Quantitative RT-PCR

Cultured cells were lysed in RNAiso Plus, and total RNA was extracted and converted to cDNA using the reverse transcription kit (Takara). Real-time PCR was performed using SYBR Select (Takara) on a QuantStudio six Flex machine (Bio-red). The primers used in qPCR were found in Table 1.

**TABLE 1:** Sequences of all primers.

Primers	Sequence (5′ to 3′)
E-cadherin-Forward	CTG​ATT​CTG​CTG​CTC​TTG​CTG​TTT​C
E-cadherin-Reverse	GGT​CCT​CTT​CTC​CGC​CTC​CTT​C
Vimentin-Forward	GCT​TCA​GAG​AGA​GGA​AGC​CG
Vimentin-Reverse	AAG​GTC​AAG​ACG​TGC​CAG​AG
MMP9-Forward	AGT​CCA​CCC​TTG​TGC​TCT​TCC​C
MMP9-Reverse	TCT​CTG​CCA​CCC​GAG​TGT​AAC​C
N-cadherin-Forward	AGG​AGT​CAG​TGA​AGG​AGT​CAG​CAG
N-cadherin-Reverse	TTC​TGG​CAA​GTT​GAT​TGG​AGG​GAT​G
β-catenin-Forward	GGC​TCT​TGT​GCG​TAC​TGT​CCT​TC
β-catenin-Reverse	CTT​GGT​GTC​GGC​TGG​TCA​GAT​G
GSK3β-Forward	AAC​TAC​CAA​ATG​GGC​GAG​ACA​CAC
GSK3β-Reverse	CCG​AGC​ATG​AGG​AGG​AAT​AAG​GAT​G
zeb1-Forward	AGT​GTT​ACC​AGG​GAG​GAG​CAG​TG
zeb1-Reverse	TTT​CTT​GCC​CTT​CCT​TTC​CTG​TGT​C
β-actin-Forward	CCTGGCACCCAGCACAAT
β-actin-Reverse	GGGCCGGACTCGTCATAC

### 2.13 Double luciferase report assay and siRNA transfection

We purchased reporter plasmids of pGL3-Basic-luc or pGL3-Basic- GSK3β-luc from Jinmai biotechnology (Chongqing, China), U87MG cells were seeded in 24-well plates at 5 × 10^4^ cells/well and transfected with 1 μl of Lipofectamine 8000 (Beyotime Biotechnology), and 0.5 μg of reporter plasmids of pGL3-Basic-luc or pGL3-Basic- GSK3β-luc in 25 μl of Opti-MEM. After 24 h of incubation, the cells were treated with quercetin or DMSO for 48 h and then harvested for luciferase assay. The luciferase reporter assay was performed with a Dual Luciferase Reporter Gene Assay Kit (RG027, Beyotime Biotechnology) according to the manufacturer’s protocol. The relative luciferase activity was calculated. Small interfering RNA (siRNA) targeting GSK3β was purchased from Sangon biotechnology (Shanghai, China), U87MG and CHG-5 cells were transfected with siRNA and cultured for 48 h before performing the assays according to the manufacturer’s instructions.

### 2.14 Xenograft mice model

Male nude mice, 4–6 weeks old, were obtained from the Experimental Animal Center of Chongqing Medical University, and the experiments were performed in accordance with the National Guidelines for Animal Care and Use Committee. The nude mice were inoculated subcutaneously with U87 cells (5 × 10^6^/0.2 ml cells per mouse) to establish the xenograft model. And then randomly divided into three groups (*n* = 6 per group). As reported, compared to the control group, treated with quercetin (100,200 mg/kg), the survival rate for mice bearing tumors was significantly higher, and no significant difference in the survival rate of the group treated with quercetin at 50 mg/kg (Hashemzaei et al., 2017). After tumor volume reached to 100mm^3^, mice were treated with quercetin (50,100 mg/kg, intraperitoneally), TMZ (50 mg/kg, intraperitoneally), or equal volume of vehicle, respectively. TMZ treatment serves as the positive control, since TMZ is the most widely used chemotherapy for patients with glioblastoma (GBM), and can extend patient’s post-operative survival. The mice were monitoring every 3 days for signs of tumor growth and body weight. Volume was calculated as (width/2)^2^ × (length/2). After 3 weeks, all mice were sacrificed, and tumor tissues were excised for immunohistochemical and western blotting analyses.

### 2.15 Statistical analysis

All results were presented as mean ± standard deviation (SD). Statistical analysis was performed by using the two-tailed Student’s t-test or ANOVA at a significance level of *p* < 0.05 (GraphPad Prism 8.0, San Diego, CA, United States).

## 3 Results

### 3.1 Quercetin inhibits the proliferation of glioma cells

**FIGURE 1:**
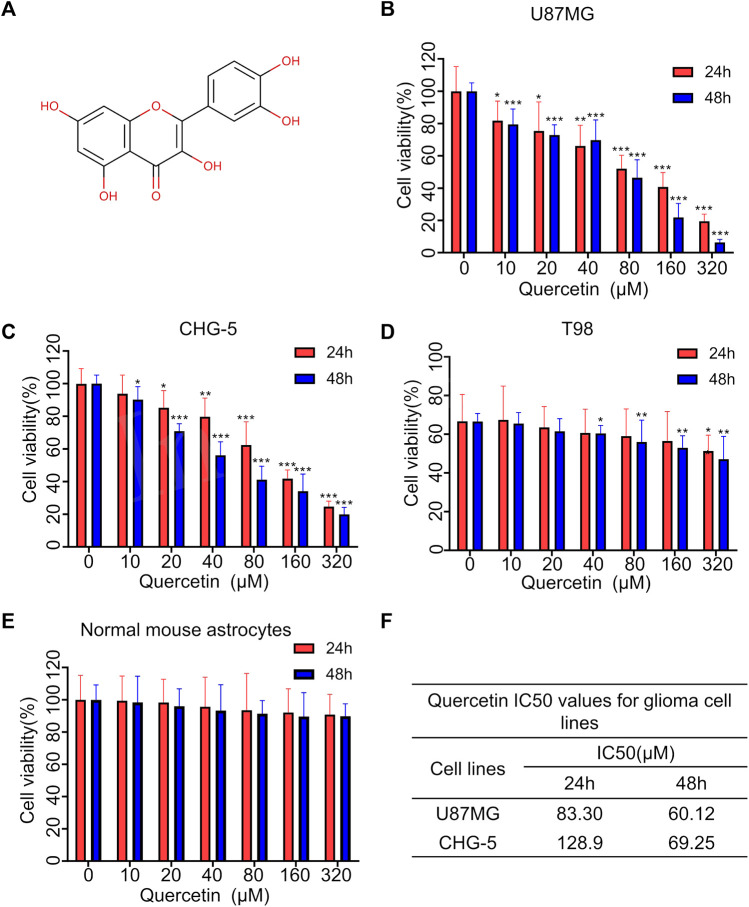
Quercetin inhibited the proliferation of glioblastoma cells *in vitro*. **(A)** The chemical structure of Quercetin. **(B–D)** Three glioma cell lines U87MG, CHG-5 and T98 were treated with various concentrations of Quercetin for 24 and 48 h, respectively, then the cell viability was measured with CCK-8. **(E)** Cytotoxic effects of quercetin. Normal mouse astrocytes were treated with various concentrations of quercetin for 24 and 48 h, then the cell viability was measured with CCK-8. **(F)** Quercetin IC50 values for glioma cell lines. All data are shown as the mean ± SD of six experiments. ^*^ *p*＜*0.05,* ^**^ *p*＜*0.01* or ^***^ *p*＜0.0*01 vs.* control group.

The chemical structure of quercetin is shown in Figure 1A. After treatment with different concentrations of quercetin for 24 and 48 h, the cell viability of three glioma cell lines U87MG, CHG-5 and T98 was evaluated using CCK-8 assay. As shown in Figures 1B–D, the cell viability of all three tested glioma cell lines was inhibited by quercetin in dose- and time-dependent manners. Compared to U87MG and CHG-5 cells, T98 cells were less sensitive to quercetin. We also confirmed that quercetin significantly decreased the cell viability of U87MG and CHG-5 cells after the cells were treated with different concentrations of quercetin for 72 h and 5 d (Supplementary Figure S1). As contrast, there was no significant effect of quercetin on normal mouse astrocytes cell viability as shown in Figure 1E. IC50 values of quercetin for U87MG and CHG-5 cells were presented in Figure 1F. These results show quercetin could significantly inhibit glioma proliferation with low toxicity.

### 3.2 Quercetin inhibits spontaneous movement of glioma cells

**FIGURE 2:**
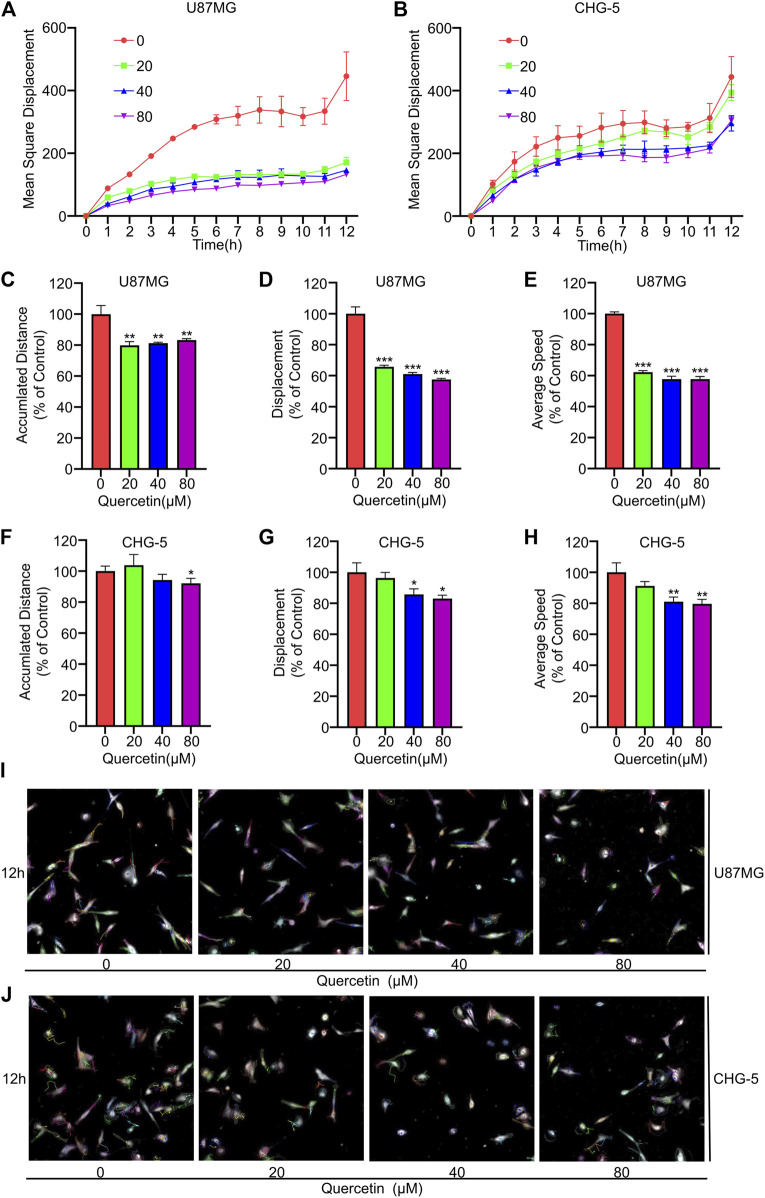
Effect of Quercetin on spontaneous movement of glioblastoma cells using high content analysis. U87MG and CHG-5 cell lines were incubated with various concentrations of Quercetin (0, 20, 40, 80 μM) for 12 h in serum free medium. Digital phase contrast (DPC) images are taken every 1 h on PerkinElmer Operetta for 12 h. Mean square displacement **(A,B)**, accumulated distance **(C–F)**, displacement **(D–G)** and average speed **(E–H)** accumulated over time for all cells were quantified and presented. Harmony 4.9 software was used to track cell displacement, and representative cell tracking images **(I,J)** were given. All data are shown as the mean ± SD of three experiments. *p＜0.05, **p＜0.01 or ***p＜0.001 vs. control group.

The effect of quercetin on U87MG and CHG-5 cells spontaneous movement was assessed using high-content imaging technology. Four cell motility parameters, including the mean square displacement, the accumulated distance, displacement and average speed, were analyzed with Harmony 4.9 software. The mean square displacement of U87MG and CHG-5 cells was showed a decreasing trend after treated with increasing concentrations (0, 20, 40, 80 μM) of quercetin for 12 h (Figures 2A,B). Similarly, the accumulated distance, displacement and average speed of U87MG and CHG-5 cells were also significantly decreased (Figures 2C–H). The cells were imaged in the digital phase contrast (DPC) channel and then tracked using DPC images. The results showed that the migration ability of the cells decreased significantly after treatment with the increasing concentrations of quercetin. (Figures 2I,J). Therefore, these data suggest that the quercetin treatment inhibits the spontaneous movement of U87MG and CHG-5 cells.

### 3.3 Quercetin inhibits glioma cells migration and invasion

**FIGURE 3:**
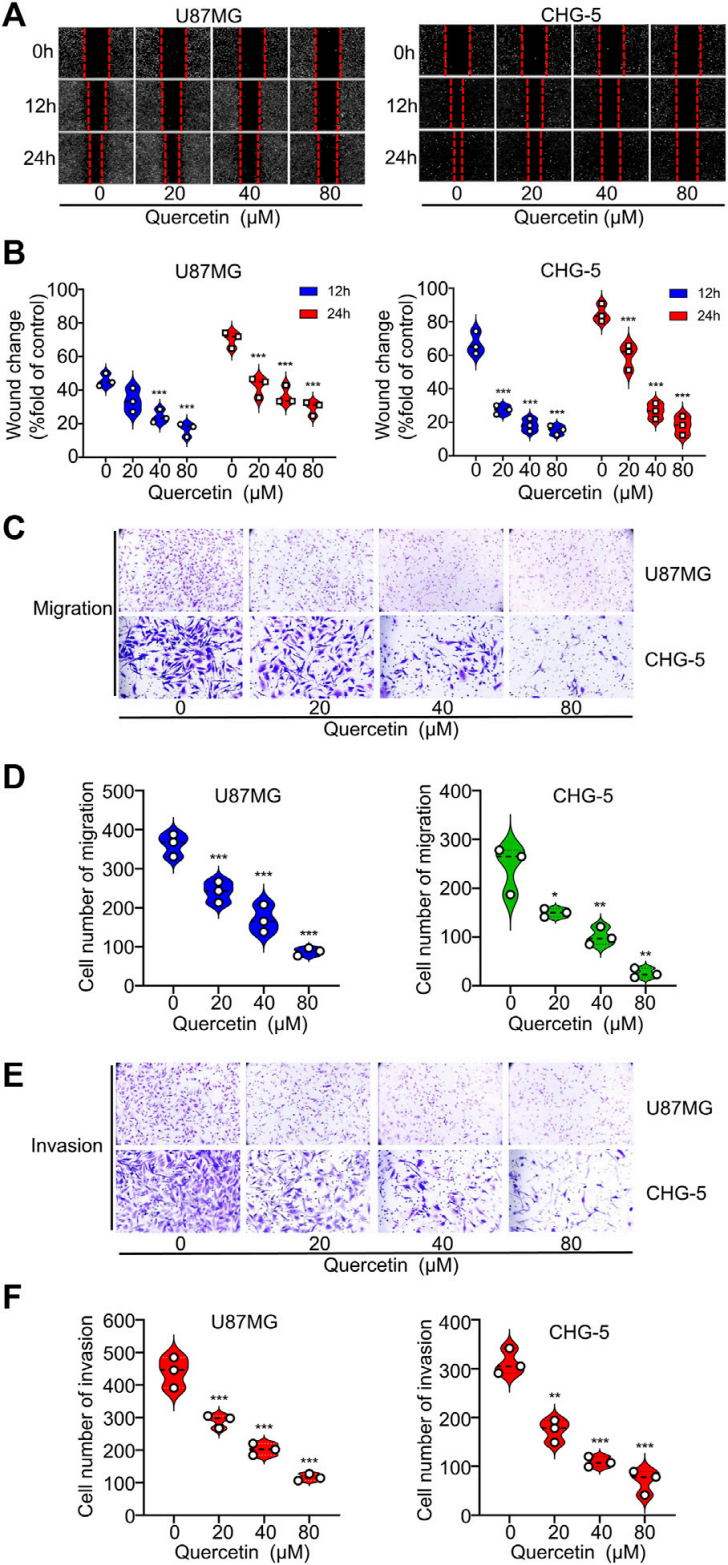
Quercetin suppresses the migration and invasion of glioblastoma cells *in vitro.* **(A)** The migration ability of U87MG and CHG-5 cells was evaluated by wound healing assay. **(B)** Quantification of the wound closure area. **(C)** Cell migration capacity of U87MG and CHG-5 cell lines was detected by transwell assay. **(D)** Quantification of the number of migrated cells. **(E)** Cell invasion ability of U87MG and CHG-5 cell lines was detected by transwell assay. **(F)** Quantification of the number of invasive cells. All data are shown as the mean ± SD of three experiments. *p＜0.05, **p＜0.01 or ***p＜0.001 vs. control group.

To further explore the effects of quercetin on the cell motility of U87MG and CHG-5, we used wound-healing assays to evaluate cell migration and performed transwell assays to assess cell invasion. The results indicated that quercetin markedly suppressed the wound healing ability of U87MG and CHG-5 cells in a dose-dependent manner (Figures 3A,B). Consistent with the wound healing assay, the transwell assay indicated that quercetin significantly suppressed the migration ability of glioma cells (Figures 3C,D). To further confirm the above results, we investigated the effect of quercetin on the invasive ability of U87MG and CHG-5 cells by transwell matrigel invasion assays. As shown in Figures 3E,F, quercetin significantly reduced the invasive capacities of U87MG and CHG-5 cells. Therefore, these present results show that quercetin suppressed migration and invasion of glioma cells.

### 3.4 Quercetin suppresses the EMT-like programme in glioma cells

**FIGURE 4:**
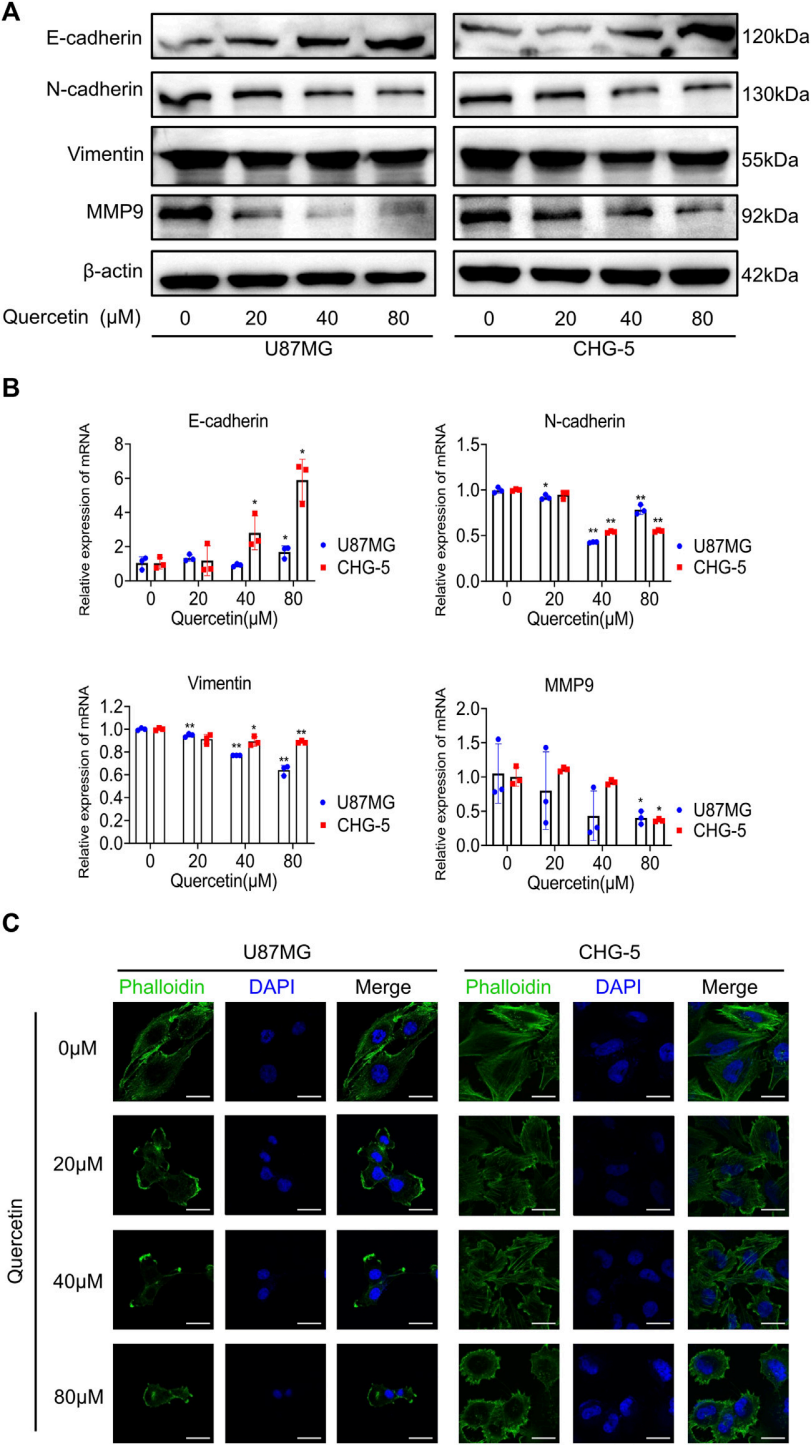
Quercetin suppresses the EMT-like programme in glioblastoma cells. **(A)** Western blotting was used to detect the expression of mesenchymal markers (N-cadherin, vimentin, and MMP-9) after treatment with quercetin (0, 20, 40, 80μM) in U87MG and CHG-5 cell lines. **(B)** RT-qPCR analysis of the mRNA levels of E-cadherin, N-cadherin, Vimentin, and MMP-9 after U87MG and CHG-5 cells were treated with quercetin (0, 20, 40, 80** **μM). All data are shown as the mean ± SD of three experiments. *p＜0.05, **p＜0.01 *vs.* control group. **(C)**Representative immunofluorescence images of the cytoskeleton (green) of U87MG and CHG-5 cells treated with quercetin (0, 20, 40, 80** **μM). Scale bar, 20 μm.

EMT is an important cellular programme for malignant progression in GBM. Since quercetin inhibits the invasive ability of GBM, we speculated that quercetin might target the EMT process of GBM. Firstly, to test the hypothesis, we examined the expressing of EMT-related markers such as E-cadherin, N-cadherin, vimentin, and MMP-9 in U87MG and CHG-5 cells. Both Western blotting and RT-qPCR results showed that quercetin decreased the expression levels of N-cadherin, vimentin and MMP-9, while increased the expression of E-cadherin in a dose-dependent manner in U87MG and CHG-5 cells (Figures 4A,B). Secondly, to further explore the effects of quercetin on EMT-like mesenchymal transition, we examined the cytomorphology of U87MG and CHG-5 cells using cytoskeletal markers (FITC-phalloidin). After treatment with different concentrations of quercetin, a significant decrease in filopodia formation was observed and cells were more round in U87MG and CHG-5 cells. (Figure 4C). Together, our data suggest that quercetin suppresses the mesenchymal transition of glioma cells.

### 3.5 Interaction of quercetin with GSK 3β

**FIGURE 5:**
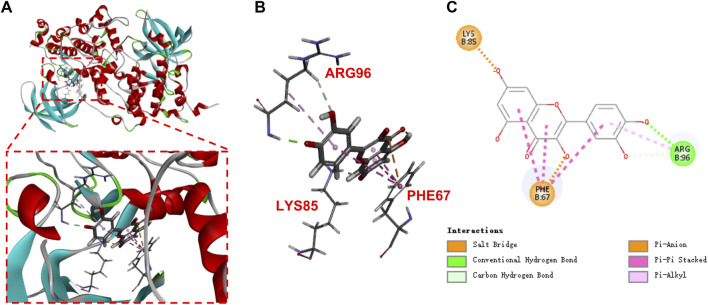
Molecular docking of quercetin **(A,B)**, 3D; **(C)**, 2D in the binding pocket of GSK3β (PDB:1H8F).

Molecular-protein docking of quercetin and GSK3β (PDB ID: 1H8F) was further conducted. Quercetin interacted with GSK3β via carbon hydrogen bond and conventional hydrogen bond and Pi-alkyl bond (ARG 96), pi-pi stacked and pi-anion bond (PHE 67), and salt bridge bond (LYS 85). The negative CDOCKER interaction energy scores between them were 47.9205 kJ/mol (Figures 5A–C), indicated that quercetin binds strongly to GSK3β.

### 3.6 Quercetin suppresses GSK3β/β-catenin/ZEB1 signaling in Glioblastoma

**FIGURE 6:**
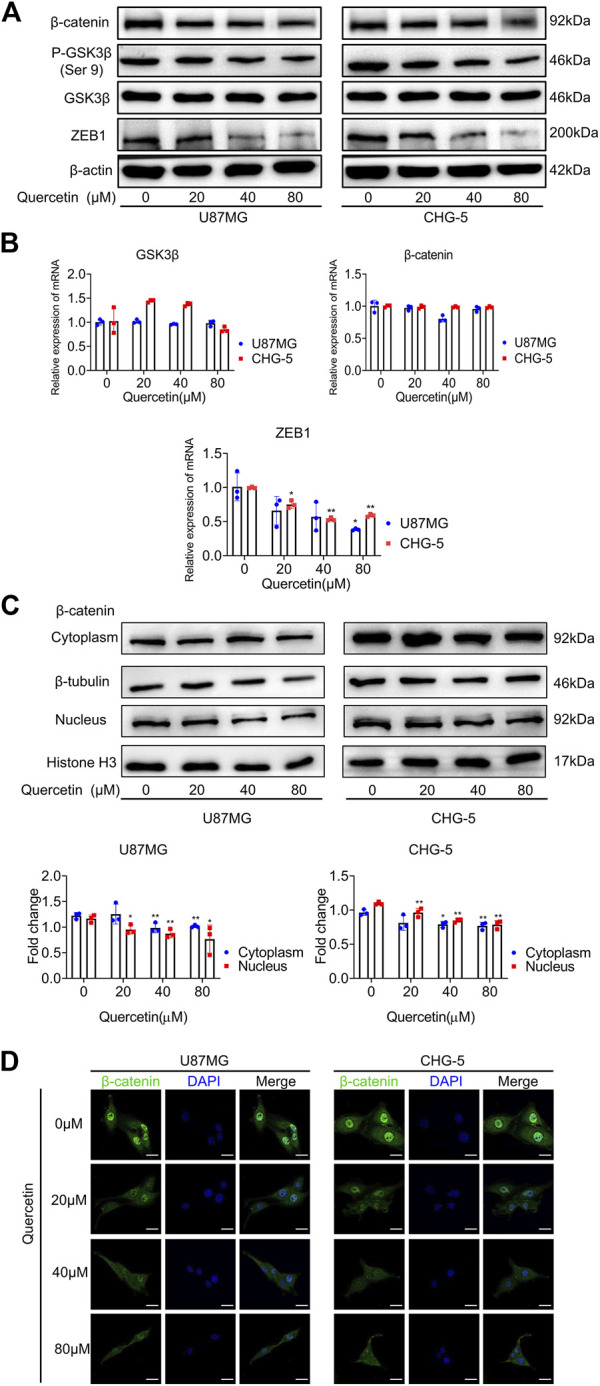
Quercetin suppresses GSK3β/β-catenin/ZEB1 pathway in GBM. **(A)** Western blotting analysis of the expression of p-GSK-3β (Ser 9), GSK-3β, β-catenin, and ZEB1 after treatment with quercetin (0, 20, 40, 80 μM) in U87MG and CHG-5 cell lines. The data are shown as the mean ± SD of three experiments. **(B)** RT-qPCR was used to examine the mRNA levels of GSK-3β, β-catenin, and ZEB1 after treatment with quercetin (0, 20, 40, 80 μM) in U87MG and CHG-5 cell lines. All data are shown as the mean ± SD of three experiments. *p＜0.05, **p＜0.01 v*s.* control group. **(C)**Western blotting was used to detect the expression of β-catenin in cytoplasm and nucleus of U87MG and CHG-5 cell lines treated with quercetin (0, 20, 40, 80 μM). The data are shown as the mean ± SD of three experiments. *p＜0.05, **p＜0.01 vs. control group. **(D)** Immunofluorescence images to exhibit the distribution of β-catenin in U87MG and CHG-5 cell lines when treated with quercetin (0, 20, 40, 80μM). Scale bar, 20 μm.

Previous findings have indicated that WNT/β-catenin signal axis and ZEB1 have a crucial role in EMT-like mesenchymal transition in GBM. To investigate whether quercetin inhibits the EMT-like mesenchymal transition of GBM *via* GSK3β/β-catenin/ZEB1signialing pathway, we used western blotting assays to assess the change of these signaling pathway proteins upon quercetin treatment. After treatment with various concentrations of quercetin, a markedly decrease in the protein levels of p-GSK-3β (Ser 9), β-catenin, and ZEB1 was observed in U87MG and CHG-5 cells (Figure 6A). RT-qPCR results showed that the mRNA level of ZEB1 was significantly decreased after quercetin treatment, while the mRNA levels of GSK-3β and β-catenin were not significantly changed (Figure 6B), suggesting that quercetin does not regulate the expression of GSK-3β and β-catenin at transcription level. By using a luciferase reporter containing GSK3β promoter, we found that quercetin did not affect the GSK3β promoter activity in U87MG cell (Supplementary Figure S2), further supporting that quercetin does not affect GSK3β transcription. In addition, our results showed that both cytoplasmic and nuclear fractions of β-catenin were decreased upon quercetin treatment. (Figure 6C). Immunofluorescence microscopy results confirmed that quercetin reduced the expression of β-catenin, especially nuclear β-catenin, in both U87MG and CHG-5 cells suggesting that quercetin might reduce nuclear translocation of β-catenin (Figure 6D). Since Ser9 is the site phosphorylated by Akt, and the phosphorylation of this residue leads to the deactivation of GSK3β. So, we examined Akt signaling following quercetin treatment. The results showed that quercetin had no significant effect on the expression levels of Akt and p-Akt (Supplementary Figure S3), implying that quercetin regulates GSK-3β signaling not through Akt.

### 3.7 GSK-3β/β-catenin/ZEB1 signaling mediates quercetin inhibition of EMT-like mesenchymal transition and invasion

**FIGURE 7:**
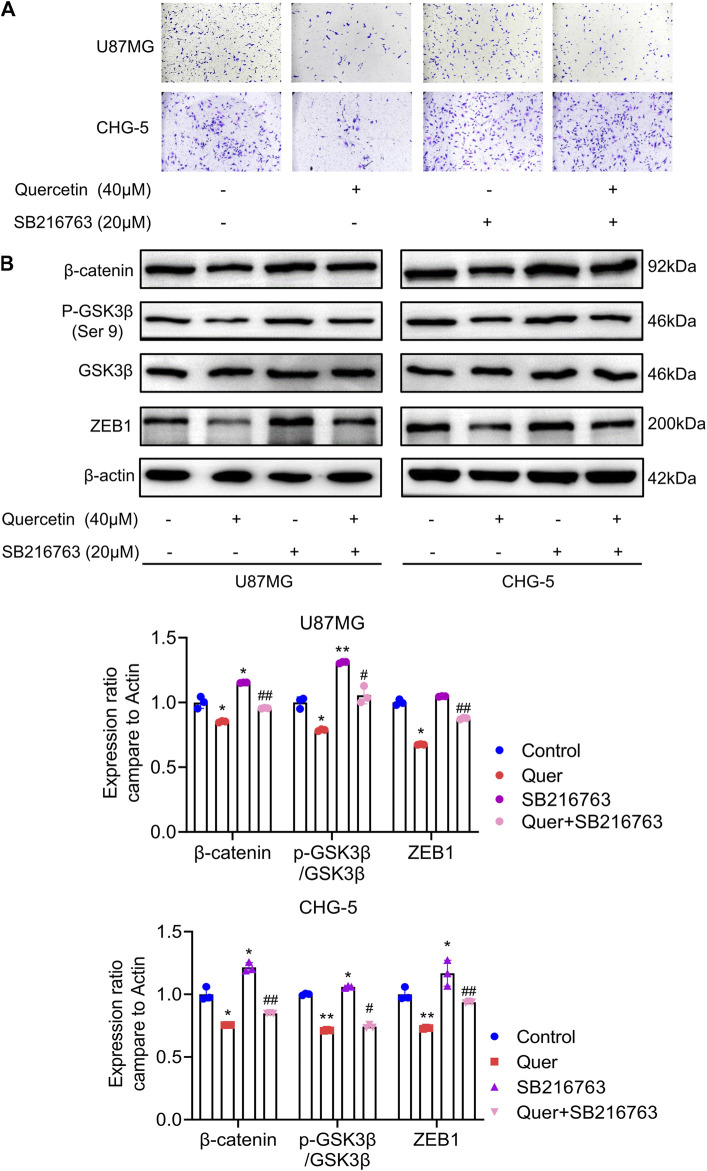
Quercetin inhibits EMT-like mesenchymal transition and invasion through GSK-3β/β-catenin/ZEB1signaling. **(A)** The cell invasion ability of U87MG and CHG-5 cells was assessed by transwell assay after treatment with quercetin alone or combined with SB216763. **(B)** Western blotting analysis of the expression of p-GSK-3β (Ser 9), GSK-3β, β-catenin, and ZEB1 after treatment with quercetin alone or combined with SB216763 in U87MG and CHG-5 cells. The data are shown as the mean ± SD of three experiments. *p＜0.05, **p＜0.01 *vs.* control group.

It has been reported that WNT/β-catenin and ZEB1 play an essential role in glioma invasion and EMT. As shown above, quercetin negatively regulates GSK-3β/β-catenin/ZEB1 signaling in GBM. Therefore, to further explore the molecular mechanism by which quercetin inhibits the cell motility of U87 MG and CHG-5, we performed transwell assays with quercetin and SB216763 (a selective GSK-3β inhibitor). The transwell invasion assays result indicated that GSK-3β inhibited by SB216763 reversed the suppression of invasive ability by quercetin in U87 MG and CHG-5 cells (Figure 7A). Furthermore, we showed that the treatment of SB216763 relieved the inhibition of GSK-3 β/β-catenin/ZEB1 signaling pathway induced by quercetin antagonist in U87 MG and CHG-5 cells (Figure 7B). However, once depletion of GSK-3β by siRNA in U87 and CHG-5 cells, quercetin repression of invasion was significantly compromised (SupplementaryFigure S4). In short, these data suggest that quercetin restrains GBM invasion and EMT-like mesenchymal transition by suppressing GSK-3β/β-catenin/ZEB1 signaling pathway.

### 3.8 Quercetin inhibits EMT-like mesenchymal transition and Glioblastoma invasion *in vivo*

**FIGURE 8:**
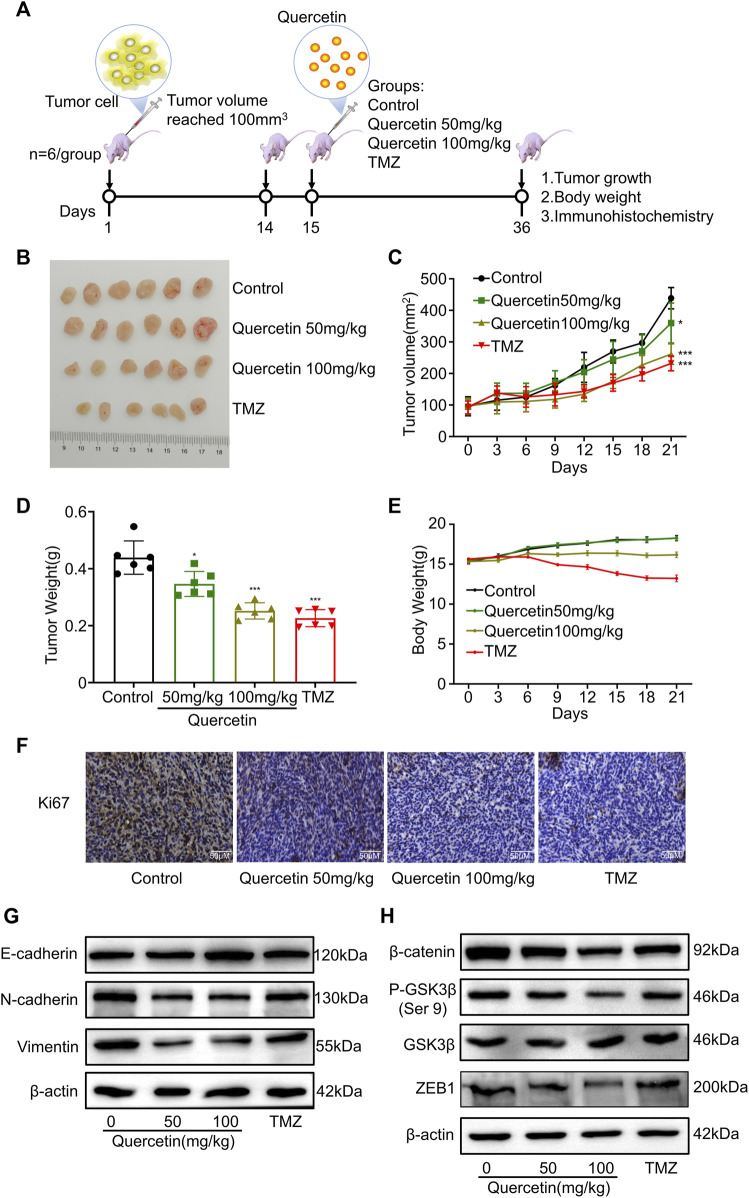
Quercetin inhibits EMT-like mesenchymal transition and GBM invasion *in vivo.* **(A)** Illustration of *in vivo* studies of xenograft mouse model. **(B)** Images of dissected xenografted tumors implanted subcutaneously with U87MG after treatment with quercetin or TMZ for 21 days. *n* = 6. **(C)** The volume of tumor was measured 3-day intervals. *n* = 6. **(D)** Tumor weight was measured at the end of the study. *n* = 6. **(E)** The body weight of mice was monitored every 3 days. *n* = 6 **(F)** Ki67 staining was detected by immunohistochemistry in tumor tissues. Scale bar = 50 μm. **(G,H)** Western blotting analysis of the expression of EMT markers (E-cadherin, N-cadherin, and vimentin) and the suppression of GSK-3β/β-catenin/zeb1 in tumor tissues. All data are shown as the mean ± SD. *p＜0.05, **p＜0.01 or ***p＜0.0*01 vs.* control group.

In order to investigate the effects of quercetin on EMT-like mesenchymal transition and GBM invasion *in vivo*, we established a xenograft mouse model. In brief, we subcutaneously implanted 2 × 10^6^ U87 MG cells into nude mice (Figure 8A). After allowing tumors to be established and tumor volume reached to 100mm^3^, all mice were treated with quercetin and temozolomide (Figure 8A). After 21 days, we found that quercetin decreased the weight and tumor volume of mice in a dose dependent manner (Figures 8B–D). In addition, compared with the control group, quercetin group had significantly less weight loss than temozolomide group. (Figure 8E). The immunohistochemical results showed that quercetin could decrease the number of Ki67-positive cells in a clear direct dose-dependent, but still higher than temozolomide group (Figure 8F). By western blotting analysis, we found that quercetin reduced the expression of N-cadherin and vimentin expression, while induced the expression of E-cadherin in subcutaneous tumor tissues (Figure 8G). Furthermore, quercetin decreased the protein levels of p-GSK-3β (Ser 9), β-catenin and ZEB1 in tumor tissue (Figure 8H). These data confirm that quercetin inhibits GBM invasion and mesenchymal transition probably *via* suppressing GSK-3β/β catenin/ZEB1 signaling.

## 4 Discussion

Glioblastoma is the most prevalent and most malignant (WHO grade IV) of primary brain tumor, with a poor overall prognosis despite aggressive multimodality therapy. Due to the low effectiveness of the current therapeutic strategies, additional drug therapy is needed on the basis of chemoradiotherapy to improve the prognosis of glioma patients. In response to this, the discovery of new high-efficiency and less-toxic therapeutic methods to inhibit the growth of cancer cells have always been the focus of research in this field.

At present, it is well known that natural products represent a type of promising anticancer drugs that inhibit tumor growth and prevent drug resistance while not toxic to normal cells (Yuan et al., 2017; Dutta et al., 2019). A growing number of phytochemicals have already been studied *in vivo* and *in vitro*, presenting development potential against malignant glioblastoma (El Gueder et al., 2018; Tavana et al., 2020; Kim et al., 2021; Do et al., 2022). Of which, quercetin, a Chinese herbal medicine, is a hopeful and efficient natural chemical product for treatment of glioblastoma. A acute toxicity research revealed that no symptoms of toxicity were observed in rabbits after treated with a single intravenous dose of quercetin (100–150 mg/kg/day) (Russo et al., 2014). Another research of a 2-year rat carcinogenicity bioassay reported that no significant adverse effects was observed in rats after treated with lower doses quercetin (50–500 mg/kg/day) (Hard et al., 2007). In addition, the majority of the studies support the anti-carcinogenic and chemoprotective effects of quercetin, and increased researches have reported that quercetin as a chemopreventive drug has an anti-tumor effect on cancer cells (Dell'Albani et al., 2017; Nguyen et al., 2017; Reyes-Farias and Carrasco-Pozo, 2019). Previous researches have shown that quercetin possesses anti-tumor activities against different cancers, such as prostate cancer, oral squamous cell carcinoma, breast cancer and glioma, through inhibition of migration, invasion, or EMT process (Srinivasan et al., 2016; Liu et al., 2017; Kim et al., 2020; Lu et al., 2020). Nevertheless, the potential molecular mechanism of quercetin against glioma invasion and metastasis largely remains unexplored.

High aggressiveness is the main clinical feature of glioblastoma. The complexity of brain invasion leads to incomplete surgical resection and poor prognosis in GBM patients. Evidence supports that mesenchymal transition, cell motility and extracellular matrix remodeling contribute to invasion and metastasis in the development of GBM. EMT, an important step during metastasis process, results in loss of cell polarity, decreased cell adhesion, and increased cell motility (Marconi et al., 2021). Our present results indicated that quercetin could significantly inhibit glioma cells proliferation in a dose-dependent manner, but had no obvious effects on the viability of normal mouse astrocytes cells, implying that quercetin has high-efficiency in anticancer activity and very low toxicity. In addition, we found that quercetin could significantly reduce the motility of glioma cells by high-content imaging technology, including mean square displacement, accumulated distance, displacement and average speed. Our findings indicate that quercetin could significantly suppress the motility of glioma cell lines. EMT has important roles in specific steps of embryonic development, wound healing, tissue reconstruction, carcinogenesis and cancer metastasis. The activation of epithelial-mesenchymal transformation (EMT) is a pivotal process for epithelial-derived malignant tumor cells to gain the ability to migrate and invade during tumor metastasis. EMT is known to loss of epithelial cell markers such as E-cadherin, and up-regulation of mesenchymal cell markers such as N-cadherin and vimentin (Lamouille et al., 2014; Dongre and Weinberg, 2019). During tumor progression, EMT enhances the resistance of tumor cells to chemotherapy and radiotherapy, which predicts poor prognosis in patients with GBM. Consequently, to improve the life of GBM patients, it is critical to efficiently suppress invasion and metastasis of GBM. Our data suggested that quercetin could significantly suppress the migration and invasion of glioma cells by wound healing and transwell assays. In addition, we observed that quercetin could increase the expressing level of E-cadherin, and decrease the expression of mesenchymal markers, such as vimentin, N-cadherin and MMP-9, indicating that quercetin could suppress the EMT process of GBM.

Pervious research has showed that WNT/β-catenin levels are elevated in glioma, and it is associated with significantly shorter survival time in glioma patients. Furthermore, evidence has implied that WNT/β-catenin signaling has a crucial role in EMT process and glioma invasion. GSK3β plays a pivotal role in WNT/β-catenin signaling and is a crucial regulator of EMT. In the present research, we found that quercetin reduces the expression of p-GSK-3β (Ser 9) and β-catenin, and these effects of quercetin were reversed by SB216763, a GSK-3β inhibitor. It has been established that deactivation of GSK3β, which phosphorylated on Ser9, leads to translocation of accumulated β-catenin to the nucleus and activates EMT transcription factors such as ZEB1, Twist, and Snail (Majewska and Szeliga, 2017). Our results revealed that quercetin decreased the expression of ZEB1 and this effect could be rescued by SB216763, implying that quercetin decreases ZEB1 expression *via* GSK-3β/β-catenin axis in glioma. These findings proved an explanation for quercetin inhibits GBM invasion primarily *via* suppressing GSK-3β/β-catenin/ZEB1 pathway. Lastly, we examined whether the anticancer effects of quercetin are related to GSK-3β/β-catenin/ZEB1 pathway *in vivo*. In the xenograft mice model, quercetin treatment decreased the volume of tumors and the expressing of Ki67 in tumor tissue in a dose-dependent manner. Subsequently, we examined the protein levels of EMT markers and GSK-3β/β-catenin/ZEB1 pathway in nude mice tumor tissue. Our results showed that quercetin could decrease the expression of EMT markers N-cadherin and vimentin, increase the expression of E-cadherin and suppress GSK-3β/β-catenin/ZEB1 signaling. In addition, the mice treated with quercetin had less weight loss than those treated with temozolomide, these results indicate that quercetin has less toxicity than temozolomide. Base on the present data of *in vitro* and *in vivo*, we hypothesize that quercetin could be developed as a potential anticancer drug for glioma. In addition, although quercetin has a highly effective anti-GBM activity, there is still a long way to go to improve its poor aqueous solubility and stability so that it can better cross the blood-brain barrier (BBB). In the future, we will extend the present studies to evaluate the mechanisms by which quercetin against glioma, and we will attempt to modify the structure of quercetin to increase its aqueous solubility and stability.

## 5 Conclusion

**FIGURE 9:**
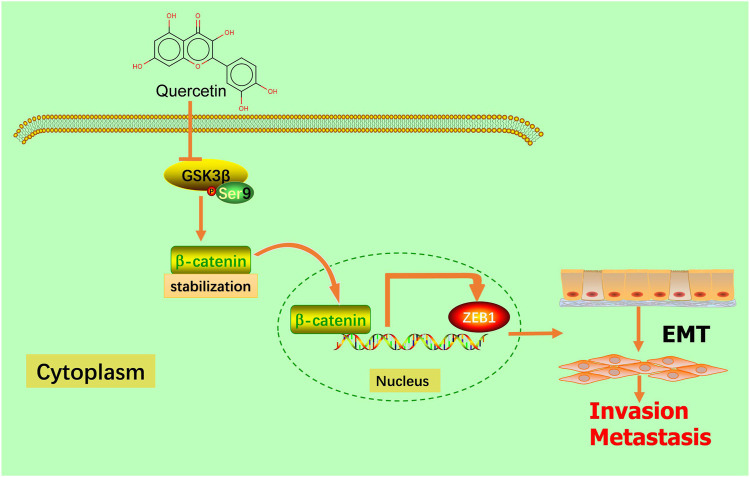
Schematic model. Quercetin suppresses invasion and metastasis *via* GSK-3β/β-catenin/ZEB1 induced EMT-like mesenchymal transition in human glioblastoma.

Our present research revealed that the natural compound quercetin could significantly suppress GBM migration and invasion *in vivo* and *in vitro* by inhibiting the EMT process *via* downregulating the GSK-3β/β-catenin/ZEB1 signaling pathway (Figure 9). In conclusion, the findings of this study suggest that quercetin is a promising therapeutic approach to treatment GBM patients in the future. Although our findings have provided new perspectives into the anti-tumor role of quercetin in glioma, further researches are necessary to describe drug targets and provide more possibilities for clinical treatment of patients with GBM.

## Data Availability

The original contributions presented in the study are included in the article/Supplementary Materials, further inquiries can be directed to the corresponding authors.
