# Supplementary figures and images for: Quercetin Suppresses Human Glioblastoma Migration and Invasion via GSK3β/β-catenin/ZEB1 Signaling Pathway
Source: Front Pharmacol. 2022 Nov 1;13:963614. doi: 10.3389/fphar.2022.963614 (PMC9663482; doi:10.3389/fphar.2022.963614)

# Figure S1

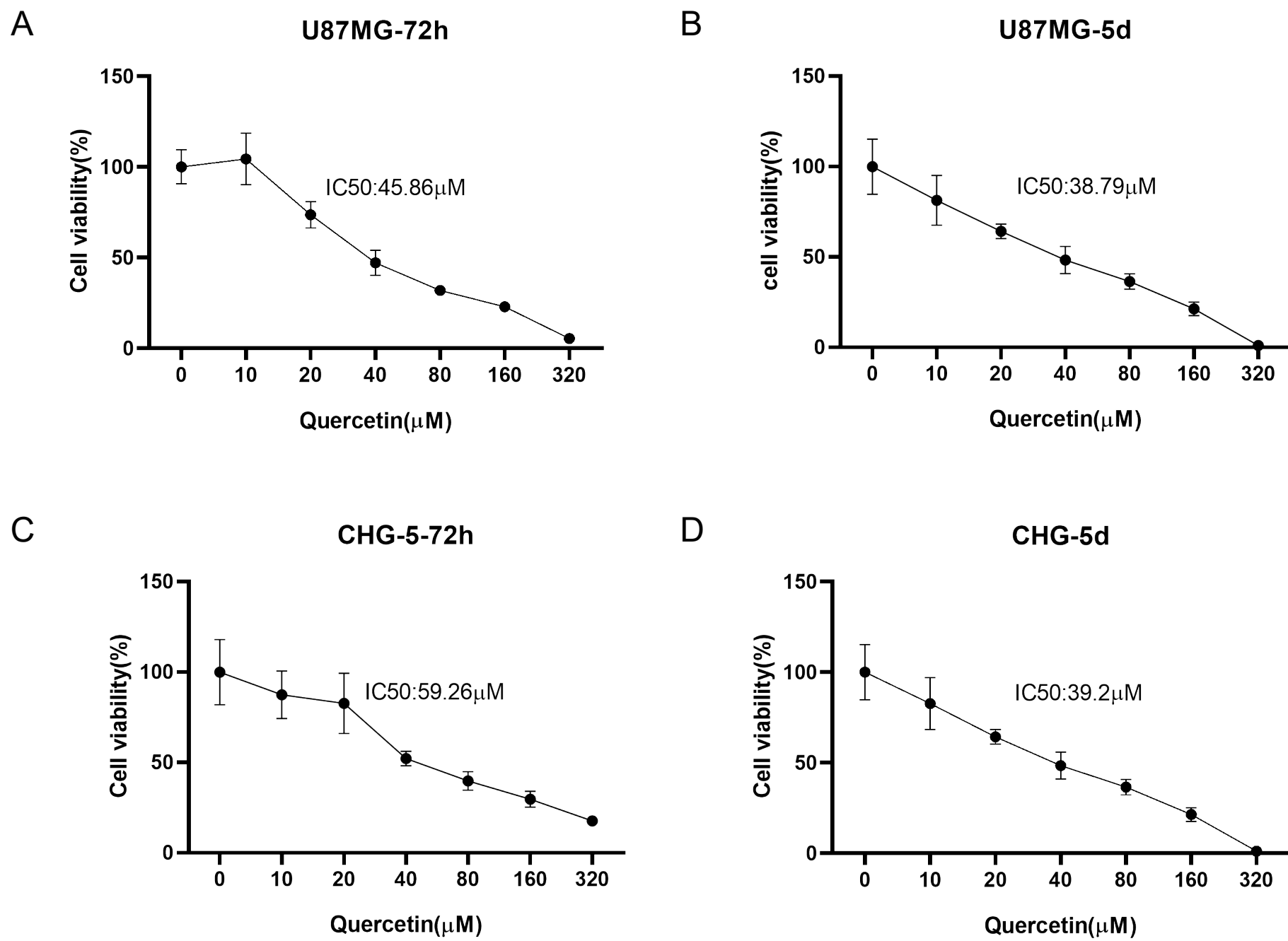

Figure S2

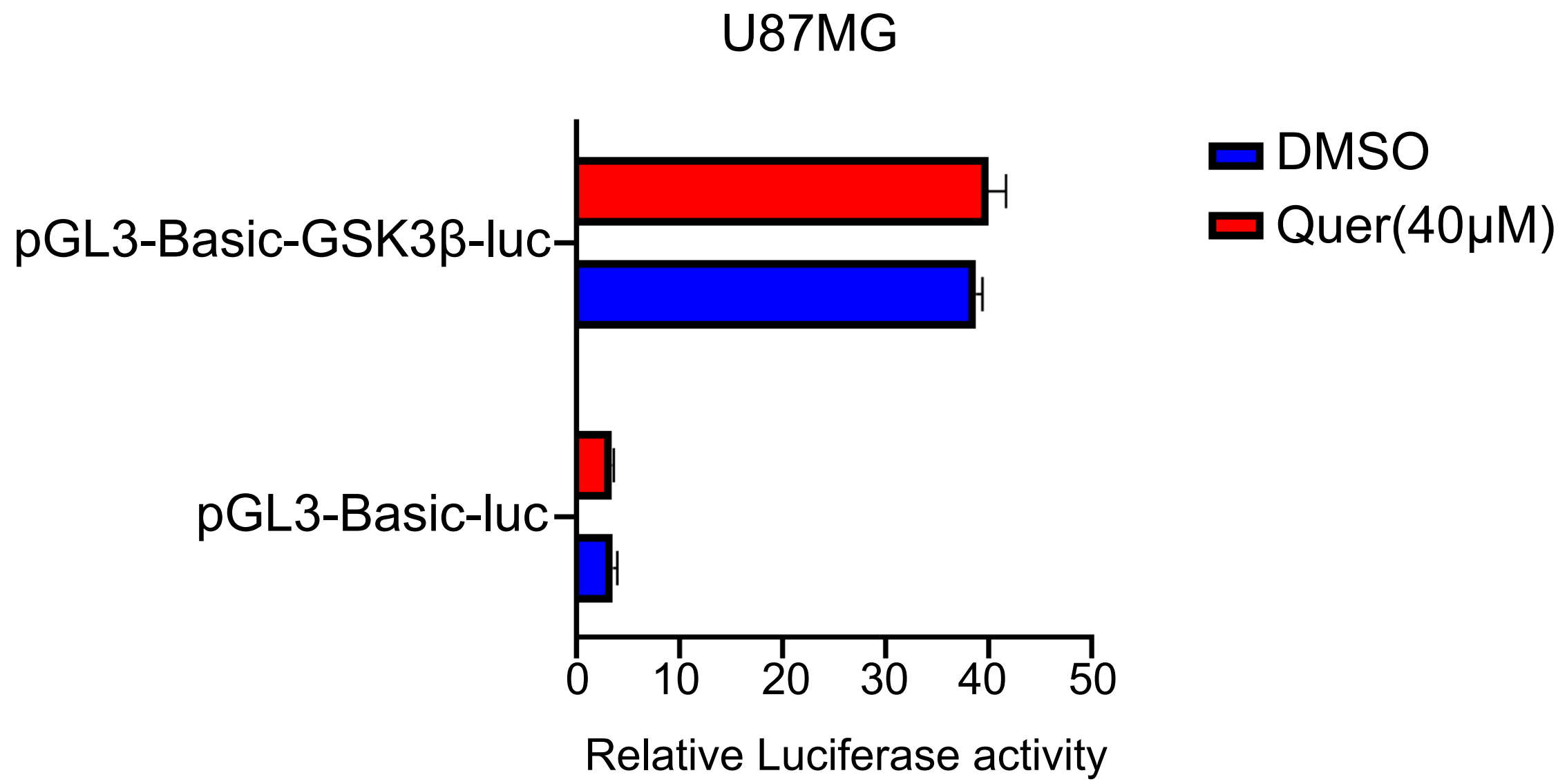

Figure S3

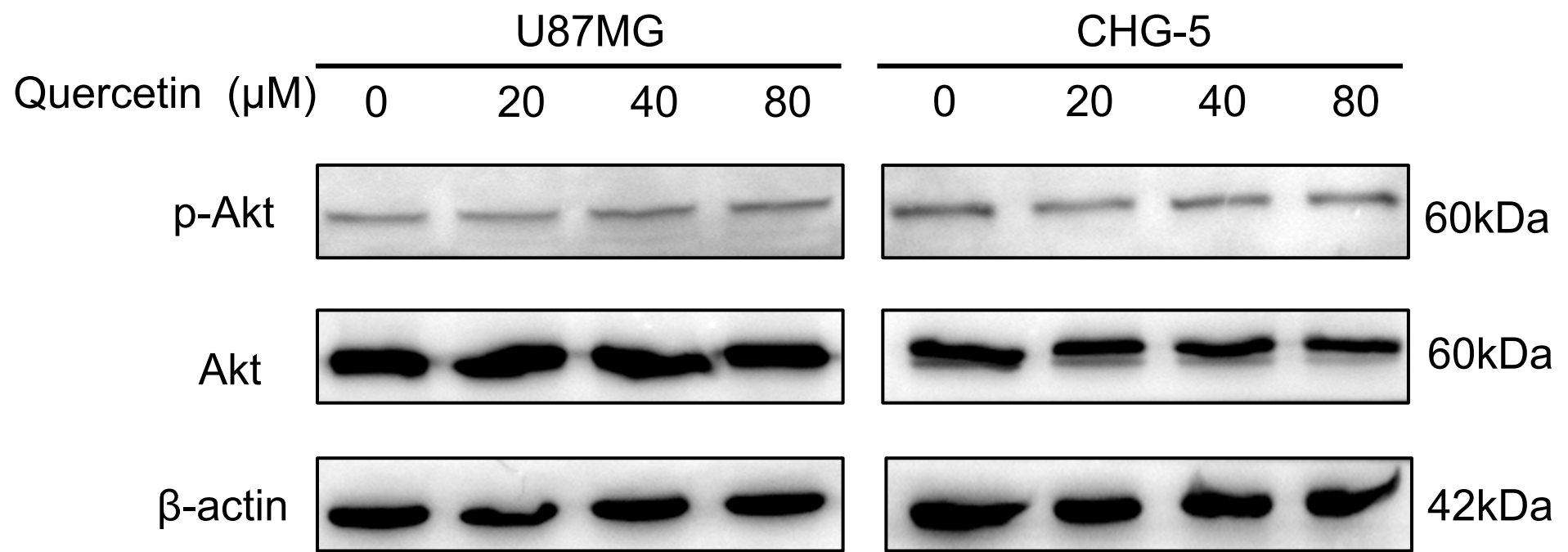

A

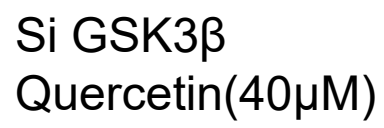

B

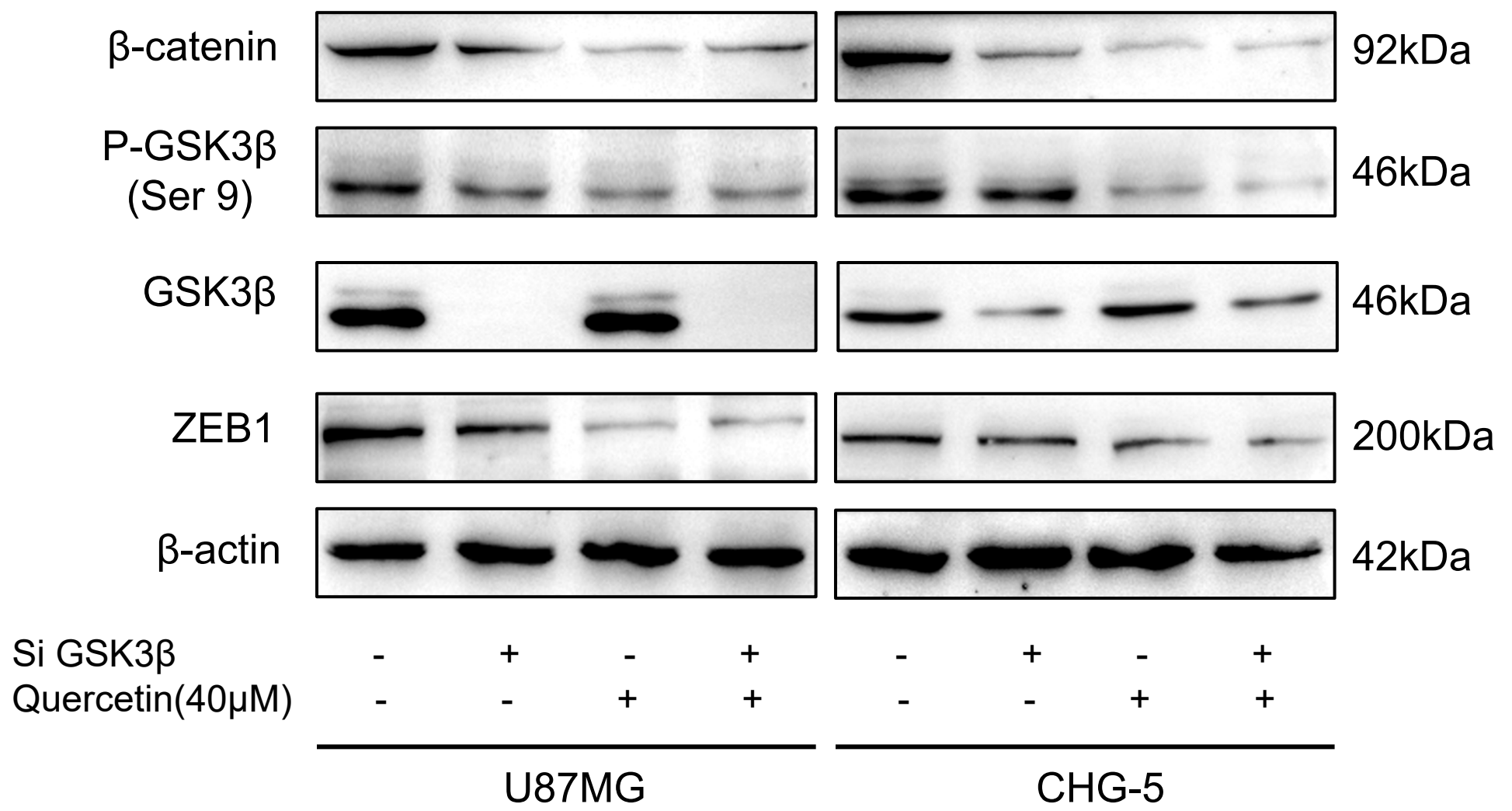

Supplement: Supplementary file 1 [file DataSheet1.pdf]
